# An AI-driven multivariate approach for personalized healthy eating recommendations aligned with sustainable healthy diet food-group guidelines

**DOI:** 10.3389/fnut.2026.1894893

**Published:** 2026-07-23

**Authors:** Dimitris Tsolakidis, Vasilis Stamatis, Lazaros P. Gymnopoulos, Vanda Pózner, Diána Szakal, Alba Lara Valtueña, Iris Blázquez, Patrick S. Elliott, Lauren D. Devine, Eileen R. Gibney, Aifric M. O'Sullivan, Kosmas Dimitropoulos

**Affiliations:** 1 The Visual Computing Lab, Information Technologies Institute, Centre for Research and Technology Hellas (ITI–CERTH), Thessaloniki, Central Macedonia, Greece; 2 Environmental Social Science Research Group (ESSRG), Budapest, Hungary; 3 Corvinus University of Budapest, Budapest, Hungary; 4 Faculty of Health Sciences, Open University of Catalonia (Universitat Oberta de Catalunya, UOC), Barcelona, Spain; 5 FoodLab Research Group (2021SGR01357), Faculty of Health Sciences, Open University of Catalonia (Universitat Oberta de Catalunya, UOC), Barcelona, Spain; 6 Institute of Food and Health, School of Agriculture and Food Science, University College Dublin, Dublin, Ireland

**Keywords:** artificial intelligence, large language models, personalized nutrition, recommender systems, retrieval-augmented generation, supervised fine-tuning, weekly meal planning, weighted Euclidean distances

## Abstract

Translating nutritional recommendations into practical day-to-day meal choices remains a challenging task, particularly when personalization, nutritional adequacy, dietary diversity, allergies, seasonal availability, and food-group constraints must be simultaneously satisfied. This study presents and evaluates the PLAN'EAT Nutrition Advisor, an Artificial Intelligence (AI)-driven, expert rule-based nutrition recommendation system, designed to generate personalized and nutritionally balanced weekly meal plans aligned with established dietary guidelines and food-group recommendations derived from Sustainable Healthy Diet (SHD) principles. The proposed approach is built upon the PLAN'EAT Expert-Curated Meal Database, a nutritionist-designed repository introduced in this work, comprising 401 expert-curated meals spanning Irish, Spanish, and Hungarian cuisines. Meal-plan generation follows a four-stage pipeline: (1) *meal filtering* based on country-specific cuisine, seasonality, dietary preferences, and allergies, (2) *daily meal plan generation* through large-scale sampling and scoring against expert-defined nutritional targets, (3) *weekly meal plan assembly* and optimization under nutritional and food-group constraints, and (4) *diversity optimization* to promote dietary variety while preserving nutritional validity. The proposed approach was validated through a large-scale *in-silico* validation involving 1,000 synthetic user profiles and the generation of 16,000 weekly meal plans corresponding to 112,000 daily meal plans. Adherence to daily and weekly nutritional targets, food-group constraints, and overall meal-plan validity at scale were assessed. In addition, we performed a preliminary descriptive evaluation against state-of-the-art Large Language Model (LLM)-based approaches under two complementary settings: Retrieval-Augmented Generation (RAG) and Supervised Fine-Tuning (SFT). Experimental results demonstrate that the proposed system can efficiently generate personalized, nutritionally compliant, and diverse weekly meal plans while maintaining transparent expert-rule-driven optimization. Furthermore, the descriptive evaluation against LLM approaches suggests that the proposed system achieves more consistent energy and macronutrient adherence under the evaluated conditions.

## 1 Introduction

Growing evidence linking diet, obesity, and non-communicable diseases (NCDs) has increased the emphasis on adopting healthier lifestyles. In particular, analyses link dietary patterns to adolescent overweight and obesity (1), while global reviews characterize its epidemiology and pathogenesis (2). The World Health Organization (WHO) estimates that at least 2.8 million deaths annually are attributable to overweight or obesity and that ~2.3% of global Disability-Adjusted Life Years (DALYs) are linked to excess weight (3). NCDs (e.g., cardiovascular disease, diabetes) account for 71% of deaths, many of which are preventable by addressing shared risk factors such as unhealthy diets (4).

At the same time, the shortcomings of a “one-size-fits-all” approach to nutrition have become evident, as individuals vary widely in their physiology, preferences, culture, health status, and behavior. These challenges have spurred the development of personalized nutrition, delivered by clinicians and increasingly supported by digital tools. Evidence from a large personalized nutrition intervention delivered online shows that personalized nutrition advice is effective (5). Building on traditional approaches and the rapid advancement of Artificial Intelligence (AI) and Machine Learning (ML) in food and nutrition (6), recommender systems can now tailor diets using anthropometric data, dietary choices, health conditions, personal preferences, real-time behaviors (e.g., dietary intake, adherence), and sensor streams (e.g., physical activity, continuous glucose monitoring) (7–11). Broadly, food and nutrition recommenders fall into two families: *traditional* and *AI-based* (12).

*Traditional systems* draw on combinatorial optimization (e.g., knapsack, integer/linear programming), content-based filtering, collaborative filtering, and hybrids. Combinatorial analysis supports meal planning by balancing nutrition, cost, and user preferences and by sequencing meals to respect constraints (13, 14). In particular, optimization methods such as the knapsack algorithm, integer programming, and constraint satisfaction are used to select optimal sets of foods while enforcing dietary diversity, food-group rules, and user restrictions (15). Although such methods can yield mathematically optimal plans, they often struggle to accommodate rich user goals, detailed rules, and diversity at scale (16, 17). Other traditional recommenders rely on content-based, collaborative, or hybrid techniques (18, 19). Content-based approaches match items to user- or item-side attributes (20, 21), collaborative methods infer preferences from similar users (22), and hybrid approaches combine both to improve accuracy and coverage (23).

However, user-item data sparsity, limited preference signals, explainability gaps, and the difficulty of encoding complex clinical guidance limit traditional systems (24). These limitations have led to the development of *AI-based* approaches. These approaches either (i) encode expert knowledge as explicit rules/constraints (knowledge-based systems) or (ii) learn preferences and outcomes from data (ML-based systems) (25).

In the knowledge-based line, (26) propose a system that nudges Mediterranean users toward culturally local foods via curated databases filtered on allergies, halal status, cuisine, and seasonality; ranking by energy needs precedes diverse weekly assembly. In (27), personalization is extended with ontology (NAct) aligned to European Food Safety Authority (EFSA)/WHO guidance and fuzzy-logic filters, followed by optimization to meet macronutrient and micronutrient targets and ensure variety.

In the ML-based line, (28) couple prediction with prescription (estimating weight-loss calories from 15 features via Support Vector Regression (SVR), trees, and linear models, then prescribing expert macronutrient splits); (29) align recommendations to regional cuisines and seasonal foods to enhance adherence; and (30) formulate diet planning as multi-objective optimization to jointly maximize nutrition/health and minimize environmental impact using genetic algorithms and sequential substitution.

More recently, there has been a rapid emergence of Large Language Model (LLM)-enabled tools for personalized nutrition. An up-to-date survey on the use of ChatGPT in nutrition reports that LLMs can draft calorie- and macronutrient-aligned plans, especially when given explicit targets, but may introduce allergens, misclassify compounds, and struggle with complex comorbidities; expert rules and oversight are therefore recommended (31). Generative pipelines have coupled deep latent models (e.g., neural and sequence models) with optimization to produce plans that meet energy and macronutrient constraints, using LLMs only to expand meal databases rather than to make clinical decisions (32). Hybrid frameworks combine ML for energy estimation with LLMs for constraint parsing from free text (33). Head-to-head evaluations against NCD profiles show ChatGPT performance improves markedly when calorie targets are provided, yet knowledge-based systems still tend to deliver the most accurate plans under clinical constraints (34). Emerging LLM-enabled nutrition systems have expanded beyond simple meal-plan generation. Recent developments include causal personalization and orchestration frameworks (35), domain-specialized food and nutrition models capable of passing chef and dietetic examinations (36), multi-objective recommendation systems augmented with LLM-based explanations (37), and mobile ecosystems that integrate food recognition, dietary forecasting, hybrid recommendation strategies, and explainability features (38). Recent work has further expanded this landscape through retrieval-augmented generation (RAG) systems that integrate evidence-based nutritional guidelines and domain knowledge to generate personalized dietary recommendations (39). At a broader level, emerging agentic AI perspectives highlight a transition from static automation toward adaptive and context-aware decision support across food and nutrition systems (40). Furthermore, injecting fuzzy linguistic variables (e.g., low/medium/high for fat, sugar, salt) into prompts has been shown to improve the clarity and nutritional rigor of LLM-generated textual advice (41), while classic ML pipelines cluster foods by nutrients and classify them for condition-aware planning with SHapley Additive exPlanations (SHAP) to improve transparency and build user trust (42).

In addition to supporting human health, dietary recommendations are increasingly expected to contribute to broader sustainability objectives. Sustainable Healthy Diets (SHDs) aim to provide nutritionally adequate, culturally acceptable, and accessible dietary patterns while reducing the environmental impacts associated with food production and consumption. Consequently, modern nutrition recommendation systems are increasingly challenged to balance personalized nutritional requirements with sustainability-oriented dietary guidance.

Despite these advances, many systems emphasize only a subset of needs, such as user anthropometric characteristics, profiles, preferences, or macronutrient targets while giving limited treatment to micronutrients and comprehensive food-group rules. Moreover, few systems incorporate seasonal and local availability, align recommendations with SHD-recommended food-group patterns, or enforce dietary variety at the weekly level at scale.

To address these gaps, this work presents the PLAN'EAT Nutrition Advisor, a novel AI-driven, knowledge-based personalized nutrition recommendation system that encodes expert dietary guidelines as explicit rules and constraints. The proposed framework combines linear programming, combinatorial search, and transparent scoring mechanisms to generate balanced weekly meal plans tailored to individual user needs while preserving dietary variety. At the core of the proposed approach lies the PLAN'EAT Expert-Curated Meal Database (hereafter “Meal Database”), a nutritionist-designed repository presented in this work, comprising 401 expert-curated meals spanning Irish, Spanish, and Hungarian cuisines. Operating on this database, our pipeline filters meals by seasonality, dietary pattern, and allergies; scores daily plans against nutritionist-validated targets; assembles and scales plans to the weekly level with additional rules; and finally enforces diversity. To comprehensively validate the proposed approach, we conducted a large-scale *in-silico* validation on 1,000 synthetic user profiles to assess adherence to daily and weekly nutritional targets. In a second experiment, we conducted a descriptive evaluation against state-of-the-art retrieval-augmented and fine-tuned LLM-based approaches to examine nutritional adherence and rule attainment under the same meal-planning task. This work contributes a unified weekly meal-planning framework that jointly incorporates nutritional, dietary, seasonal, allergy, and diversity constraints, together with the introduction of the PLAN'EAT Expert-Curated Meal Database and a large-scale *in-silico* validation across diverse user profiles, seasons, and meal-database configurations. The framework is further evaluated against retrieval-augmented and fine-tuned LLM-based approaches.

The remainder of the paper is organized as follows. Section 2 presents the Meal Database and details the proposed AI-based recommendation framework. Section 3 presents the experimental evaluation, including the *in-silico* study on user profiles and a head-to-head comparison with retrieval-augmented and fine-tuned LLM baselines. Section 4 discusses the results and limitations, and Section 5 concludes the paper.

## 2 Materials and methods

In this section, the methodological framework and underlying assumptions of the PLAN'EAT Nutrition Advisor are presented. We first describe the construction and organization of the Meal Database, including the formalization of food-items, dishes, and meals for nutritionally consistent meal composition, and the derivation of their nutrient and food-group attributes (Section 2.1). We then present the recommendation methodology (Section 2.2), including data initialization and aggregation, user profiling and target calculation, candidate meal filtering, and the generation, scoring, and selection of daily and weekly plans.

### 2.1 The PLAN'EAT expert-curated meal database

The Meal Database is an expert-curated resource designed to support the generation of personalized, nutritionally balanced meal plans. It integrates standardized food composition data with nutritionist-designed dishes and meals, structured to enable consistent evaluation of nutritional and dietary constraints.

#### 2.1.1 Meal database design

The Meal Database comprises three layers—*food-items, dishes*, and *meals*–that underpin the entire recommendation system. The Meal Database is supported by McCance and Widdowson's *The Composition of Foods Integrated Dataset 2021* (43), a UK national reference with about 3,000 foods providing comprehensive composition data across macronutrients, micronutrients, and related descriptors. We selected this source for its breadth, documentation quality, and stability. The Meal Database has three levels in which food-items serve as atomic entities, dishes represent combinations of food-items with specified quantities, and meals correspond to structured collections of dishes.

##### 2.1.1.1 Food-items

Food-items include all foods from the McCance and Widdowson dataset. We use data from the Proximates, Inorganics, and Vitamins tables, selecting only the attributes required for meal planning, as summarized in Table 1.

**Table 1:** Selected attributes for food-items from McCance and Widdowson tables.

Category	Attributes
Identifiers	Food Code, Food Name, Description
Classification	Group
Energy	Energy (kcal)
Macronutrients	Protein (g), Fat (g), Carbohydrate (g)
Fiber	AOAC fiber (g)
Micronutrients	Folate (μg), Calcium (mg), Iron (mg)

The selected attributes are aligned with food-group and nutritional targets adopted from the SHD and dietary reference frameworks (e.g., EFSA). The Group attribute is a hierarchical code that assigns each food-item to progressively more specific categories (e.g., M = Meat and meat products, MC = Poultry, MCA = Chicken). It is central to the system, as it enables dietary filtering (preferences, allergies) and supports the calculation of food-group quantities and serving counts used during scoring. All nutrient values are expressed per 100 g, following the standard reporting format of the McCance and Widdowson dataset. Fiber values correspond to AOAC fiber (Association of Official Analytical Chemists), as defined in the source dataset.

##### 2.1.1.2 Dishes

A dish is a nutritionist-designed recipe composed of one or more food-items with specified quantities. Each dish record includes an identifier, a name, a concise description, and, where useful, preparation instructions. Up to ten food-items (ingredients) are linked to the dish via their corresponding codes, together with their quantities in grams, ensuring manageable recipe complexity and consistent nutritional aggregation. Food-group membership is recorded at the dish level based on the Group attribute of the constituent food-items (e.g., meat, fish, dairy, legumes, vegetables, fruit). This enables consistent representation of dish composition in downstream processing. All dishes were authored by nutritionists to ensure nutritional adequacy and culinary realism.

##### 2.1.1.3 Meals

A meal is an assembly of one to eight dishes and is categorized as Breakfast, Snack, Lunch, or Dinner. Each meal record includes an identifier, a name, an optional description, the ordered list of constituent dish identifiers, and seasonality flags (Autumn, Winter, Spring, Summer). Nutritional attributes are computed by aggregating the constituent dishes, including energy, macronutrients, and micronutrients. In addition, food-group attributes–propagated from item-level categorical labels through dish-level assignments–are recorded at the meal level as both gram-level quantities and, where applicable, standardized serving counts. At the daily level, these include vegetables, fruits, fruit juice, legumes, dairy, cheese, and nuts, while at the weekly level they additionally include meat and meat products, beef/lamb/veal, fish and fish products, and oily fish. Binary presence flags for meat, fish, dairy, and nuts are also retained.

#### 2.1.2 Meal database population and curation

Building on the defined Meal Database structure, nutrition researchers in Ireland, Spain, and Hungary populated the dish and meal tables to support complete weekly plans for healthy adults across seasons, dietary patterns, and allergies. Each country followed a structured protocol to ensure cultural relevance, nutritional accuracy, and interoperability. Database compilation began with a literature review to identify dominant dietary patterns and their associated health and environmental impacts. This was followed by a systematic search of national meal and recipe databases, generating an initial list of candidate dishes. Original recipes were then retrieved and standardized to single-person servings using national reference tables and retained only when they were both nutritionally balanced and culturally representative. Each dish was decomposed into its constituent ingredients and preparation methods, with all components coded according to the McCance and Widdowson's dataset. When an ingredient from the original recipe did not have an exact corresponding food-item in the dataset, a substitute food-item was selected by the nutrition researchers on a case-by-case basis. The selection process considered nutritional composition, food category, culinary use, sensory characteristics, and expert judgement, with the objective of identifying the closest available equivalent within the dataset. Such substitutions were infrequent and were primarily limited to culturally specific ingredients or regional ingredient variants. Seasonal availability was assigned using country-specific produce calendars. All entries underwent iterative quality control, including translation, food-item coding, and nutrient verification. Finally, the three country-level datasets were combined and harmonized to ensure a consistent structure, terminology, and allergen annotation.

Table 2 summarizes the number of meals available in each country-specific database and in the combined corpus, overall and by season and meal type.

**Table 2:** Meal catalog sizes by dataset, season, and meal type.

Season	Breakfasts	Snacks	Lunches	Dinners	Total
Hungarian					
Autumn	34	34	39	39	146
Winter	28	29	30	30	117
Spring	28	27	34	31	120
Summer	33	33	38	37	141
Total	35	35	40	40	150
Irish					
Autumn	26	25	25	25	101
Winter	26	25	25	25	101
Spring	26	25	25	25	101
Summer	26	25	25	25	101
Total	26	25	25	25	101
Spanish					
Autumn	32	25	25	27	109
Winter	31	25	25	27	108
Spring	31	27	26	32	116
Summer	30	33	30	26	119
Total	35	35	41	39	150
Combined					
Autumn	92	84	89	91	356
Winter	85	79	80	82	326
Spring	85	79	85	88	337
Summer	89	91	93	88	361
Total	96	95	106	104	401

Dataset totals differ from the corresponding column sums because meals frequently belong to multiple seasons. Seasonal totals, by construction, equal the corresponding row sums.

### 2.2 Recommendation methodology

The proposed recommendation methodology integrates user-specific constraints, expert-defined food-group rules, weighted score-based optimization over expert-defined nutritional targets, and diversity requirements to generate personalized, nutritionally balanced weekly meal plans aligned with established dietary guidelines.

Building on the Meal Database described in Section 2.1, the recommendation process begins with data initialization and aggregation, followed by filtering of eligible meals, generation and scoring of daily plans, composition and scoring of weekly plans, and final selection of a diverse weekly plan. The overall objective is to produce personalized weekly meal plans that satisfy nutritional targets, dietary constraints, and variety requirements.

**Figure 1:**
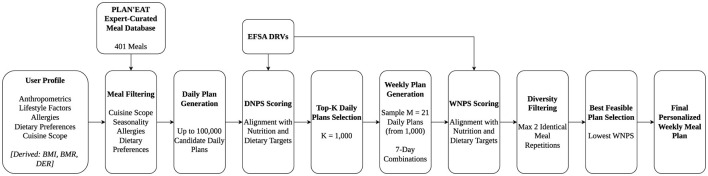
Overview of the PLAN'EAT Nutrition Advisor workflow.

Figure 1 summarizes the complete recommendation pipeline of the PLAN'EAT Nutrition Advisor, from user-profile input to final weekly meal-plan generation.

#### 2.2.1 Data aggregation for meal-level representation

To enable consistent meal-level representation for scoring and constraint evaluation, we initially aggregate nutrient and food-group information hierarchically from food-items to dishes and from dishes to meals. Nutrient values, expressed per 100 g at the food-item level, are scaled by food-item quantities and summed to obtain dish-level totals, which are then aggregated to the meal level. Food-group quantities and serving counts are accumulated across components, while binary group indicators (e.g., presence of meat or fish) are propagated using logical rules. Precomputing these per-meal attribute vectors enables efficient and consistent evaluation during plan generation and scoring.

#### 2.2.2 User profile and derived quantities

The user profile includes sex, year of birth *YB*, height *H* (cm), weight *W* (kg), Physical Activity Level *PAL* per (44), dietary preference (Omnivore, Pescatarian, Vegetarian, Vegan), allergies (Dairy; Nuts), and preferred cuisine scope, allowing users to select one or more of the available country-specific datasets (Ireland, Spain, Hungary). From the anthropometric and activity-related inputs, the system derives the user's age *A*, Body Mass Index *BMI* and Basal Metabolic Rate *BMR* per (45), and Daily Energy Requirements *DER*. The computations are given in Equations 1–5.


$$\begin{array}{l} A=Y_{\text{ref}}-YB \end{array}$$ (1)

$$\begin{array}{l} H_{m}=\frac{H}{100} \end{array}$$ (2)

$$\begin{array}{l} \text{BMI}=\frac{W}{H_{m}^{2}} \end{array}$$ (3)

$$\begin{array}{l} \text{BMR}=a\cdot W+b\cdot H\pm c \end{array}$$ (4)

where *a*, *b*, and *c* are age- and sex-specific coefficients selected according to the user's computed age *A* (Equation 1) and sex, using the values reported in column “kcal day^−1^” of Table 15 in (45).


$$\begin{array}{l} \text{DER}=\text{BMR}\times \text{PAL} \end{array}$$ (5)

#### 2.2.3 Filtering

Filtering selects the meals that are compatible with the user profile before any scoring is performed. Meals are retained only if they match the current season and comply with the user's dietary preferences, allergies, and selected cuisine scope. The result is a set of eligible breakfasts, snacks, lunches, and dinners for plan generation.

#### 2.2.4 Daily plan generation, sampling, and scoring

From the filtered meals, the full set of candidate daily plans can be expressed as all feasible ordered 5-tuples (Breakfast, Morning Snack, Lunch, Afternoon Snack, Dinner). As the number of such combinations is large, we approximate this space by uniformly sampling up to *N* = 100, 000 candidate plans per user. These are then scored using the Daily Nutrition Plan Score (DNPS), defined in Equations 6, 7 as a weighted Euclidean distance over the deviations of individual nutritional features from their corresponding targets. In Equation 6 *a*_*i*_ denotes the achieved value of feature *i* in a candidate daily plan, *t*_*i*_ denotes the corresponding target value or target interval, *w*_*i*_ denotes the feature weight, and Δ_*i*_ denotes the deviation from the target as defined in Equation 7. In Equation 7, *L*_*i*_ and *U*_*i*_ denote the lower and upper desirable bounds, respectively, for feature *i* when the target is specified as an interval, whereas τ_*i*_ denotes the desired value when the target is specified as a single point. The normalized score (NDNPS) is given in Equation 8. The feature-specific weights and targets are given in Table 3. Feature weights were defined through consultation with the nutrition experts involved in the development of the recommendation framework and were intended to reflect the relative importance of the different nutritional objectives during candidate-plan ranking. The weighting strategy was informed by established nutritional requirements and broader healthy and sustainable dietary behavior principles adopted within the PLAN'EAT project. Specifically, greater emphasis was assigned to energy adherence, followed by macronutrient balance, food-group adherence, micronutrient adequacy, and fiber adequacy. The weights were specified *a priori* and were not derived through data-driven optimization or statistical fitting. Macronutrient and micronutrient targets are set based on EFSA Dietary Reference Values (DRVs) (44), while food-group targets are adapted from SHD guidelines developed and evaluated in the MyPlanetDiet randomized controlled trial (46, 47).


$$DNPS\left(\text{a}\right)=\sqrt{\overset{m}{\underset{i=1}{\sum \text{​}}}w_{i}\Delta_{i}{\left(a_{i},t_{i}\right)}^{2}}$$ (6)

$$\Delta_{i}\left(a_{i},t_{i}\right)=\{\begin{array}{cc} 0, & t_{i}=\left[L_{i},U_{i}\right]\text{and}\;L_{i}\leq a_{i}\leq U_{i}\\ L_{i}-a_{i}, & t_{i}=\left[L_{i},U_{i}\right]\;\text{and}\;a_{i}<L_{i}\\ a_{i}-U_{i}, & t_{i}=\left[L_{i},U_{i}\right]\text{and}\;a_{i}>U_{i}\\ \left|a_{i}-\tau_{i}\right|, & t_{i}=\tau_{i}\;\left(\text{point target}\right) \end{array}$$ (7)

$$NDNPS\left(\text{a}\right)=\frac{DNPS\left(\text{a}\right)}{DNPS\left(\text{a}\right)+1}$$ (8)

Candidates are ranked in ascending order of NDNPS, so that lower values correspond to closer adherence to the target profile. To seed the weekly stage with high-quality yet diverse building blocks, we retain the top *K* = 1, 000 plans and sample uniformly *M* = 21 of them for subsequent processing.

**Table 3:** Feature targets and weights for daily/weekly plan scoring (DNPS/WNPS).

Category	Feature	Recommended Dietary Allowance (RDA)/Target		Weight		Subtotal
		Daily	Weekly	Daily	Weekly	Daily/Weekly
Energy	Energy (kcal)	DER	7 × daily	0.40		0.40	
Macronutrients	Protein (g)	[(DER × 15%)/4, (DER × 25%)/4]	7 × daily	0.12		0.24	
	Carbohydrate (g)	[(DER × 45%)/4, (DER × 60%)/4]	7 × daily	0.06			
	Fat (g)	[(DER × 20%)/9, (DER × 35%)/9]	7 × daily	0.06			
Fiber	Fiber (g)	DER/2500 × 25	7 × daily	0.06		0.06	
Micronutrients	Calcium (mg)	DER/2500 × 1000	7 × daily	0.04		0.10	
	Iron (mg)	DER/2500 × 11 (male), DER/2500 × 17 (female)	7 × daily	0.04			
	Folate (μg)	DER/2500 × 330	7 × daily	0.02			
Food groups	Vegetables (g; serv)	DER/2500 × 300 g; 3 servings	7 × daily	0.03; 0.01	0.02; 0.005	0.20	
	Fruit (g; serv)	DER/2500 × 200 g; 2 servings	7 × daily	0.03; 0.01	0.02; 0.005		
	Legumes (g)	DER/2500 × 125 g	7 × daily	0.05	0.05		
	Dairy (g; serv)	[DER/2500 × 125, DER/2500 × 250] g;1–2 servings	7 × daily	0.02; 0.005	0.01; 0.005		
	Cheeses (g; serv)	DER/2500 × 25 g; 1 serving	7 × daily	0.01; 0.005	0.005; 0.005		
	Nuts (g)	DER/2500 × 30 g	7 × daily	0.03	0.025		
	Meat and meat products (g; serv)	N/A	420 g; 3 servings	N/A	0.01; 0.005		
	Beef, Lamb, Veal (g; serv)	N/A	120 g; 1 serving	N/A	0.005; 0.005		
	Fish and fish products (g; serv)	N/A	200 g; 2 servings	N/A	0.01; 0.005		
	Oily fish (g; serv)	N/A	100 g; 1 serving	N/A	0.005; 0.005		

DER, Daily Energy Requirements.

#### 2.2.5 Weekly generation

Candidate weekly plans are constructed by selecting 7 out of the *M* = 21 retained daily plan. This yields a combinatorial space of 217, providing substantial diversity while remaining computationally tractable. The plans are then scored using the Weekly Nutrition Plan Score (WNPS), defined analogously to DNPS as a weighted Euclidean distance over per-feature deviations from their corresponding weekly targets. The feature-specific weekly targets and weights are given in Table 3, while the notation and formal definition follow Equations 6, 7. The normalized score (NWNPS) is computed as in Equation 8. Weekly targets are derived primarily by scaling the corresponding daily targets by a factor of seven, consistent with EFSA DRVs and the underlying nutritional framework. For selected food groups, fixed weekly targets are specified based on nutritionist guidance and SHD recommendations. Weekly feature weights are based on the same prioritization principles as the daily scoring stage, with additional weekly food-group objectives incorporated as shown in (Table 3). Finally, candidate weekly plans are ranked in ascending order of NWNPS, so that lower values correspond to closer adherence to the target profile.

#### 2.2.6 Diversity filtering and final suggestion

To promote dietary variety while preserving sufficient flexibility for optimization, candidate weekly plans are considered feasible only if no meal appears more than twice within the same weekly plan, i.e.,


$$\begin{array}{l} \underset{m\in W}{\max}\text{count}\left(m\right)\leq 2 \end{array}$$ (9)

where count(*m*) denotes the number of occurrences of meal *m* in weekly plan *W*. This threshold was selected empirically to discourage excessive repetition of identical meals during the week while avoiding overly restrictive constraints that could substantially reduce the number of feasible plans for users with limited meal availability due to seasonality, dietary preferences, or allergies. Feasible candidates are traversed in ascending order of NWNPS, and the first plan satisfying this constraint is selected; its rank position serves as a diversity-depth indicator. If no candidate satisfies the diversity constraint (e.g., due to seasonality, user-profile constraints, or database limitations), selection falls back to the overall ranked set, and the best-scoring plan is returned.

#### 2.2.7 Computational Performance, Runtime Requirements, and Scalability

To assess computational performance, runtime requirements, and scalability, the recommendation engine was evaluated on an OVH Cloud virtual machine equipped with 4 vCPUs (Intel Haswell architecture) and 8 GB RAM. The system was configured to use three parallel worker processes. Under these conditions, the system was found to be CPU-bound and required approximately 5-6 seconds on average to generate a weekly meal-plan recommendation. The use of multiple worker processes enabled concurrent handling of recommendation requests, indicating that throughput can be increased through parallel execution as computational resources become available.

## 3 Results

The proposed recommendation system was examined through two complementary experiments. First, we performed a large-scale *in-silico* validation using synthetic user profiles to assess adherence to nutritional rules and targets across daily and weekly meal plans. Second, we evaluated the system against state-of-the-art open-source LLM-based approaches.

### 3.1 *In-silico* validation on synthetic user profiles

#### 3.1.1 Synthetic user profile generation and validation protocol

To validate the planner under realistic variability, we simulated a large cohort of user profiles designed to approximate population-level variation in energy requirements, dietary preferences, allergies, and sex, and executed the full recommendation pipeline for each profile across the Irish, Spanish, Hungarian, and combined databases.

Daily Energy Requirements (DER) were sampled from sex-specific Normal distributions informed by prior literature (48) as defined in Equations 10, 11.


$$DER_{\text{M}}\sim N\left(2.5\times 10^{3},{\left(\frac{1}{3}\times 10^{3}\right)}^{2}\right)\text{kcal/d}$$ (10)

$$DER_{\text{F}}\sim N\left(2.0\times 10^{3},{\left(\frac{4}{14}\times 10^{3}\right)}^{2}\right)\text{kcal/day}$$ (11)

Dietary preferences (omnivore, pescatarian, vegetarian, vegan) and allergies (none, dairy, nuts, dairy & nuts) were assigned according to European prevalence estimates (49). The final distribution of synthetic user profiles used in the experiments is presented in Table 4.

**Table 4:** Number of synthetic user profiles by dietary preference, allergies, and sex.

Allergies	Dietary Preference								Total
	Omnivore		Pescatarian		Vegetarian		Vegan		
	M	F	M	F	M	F	M	F	
None	403	403	14	14	32	32	9	9	916
Dairy	24	24	1	1	2	2	0	0	54
Nuts	11	11	0	0	1	1	0	0	24
Dairy & Nuts	3	3	0	0	0	0	0	0	6
Total	441	441	15	15	35	35	9	9	1,000

Since the recommender operates on three country-specific datasets (Ireland, Spain, Hungary) as well as a combined corpus, the validation procedure was performed for each synthetic user profile across all four database configurations and for each of the four seasons. For every profile × dataset × season combination, the system generated candidate daily meal plans and subsequently constructed the corresponding seven-day weekly plan.

For each generated day and week, both the achieved values and the corresponding nutritional targets were stored for all evaluated features. Overall, the experimental protocol produced 16, 000 weekly meal plans (1,000 users × 4 seasons × 4 databases), corresponding to 112, 000 daily meal plans.

#### 3.1.2 Nutritional target adherence and validation results

The validation results are organized into three complementary dimensions: (i) adherence to energy targets, (ii) adherence to nutrient-related targets, and (iii) adherence to food-group consumption rules. Together, these analyses assess the recommender's ability to satisfy both quantitative nutritional requirements and qualitative dietary-pattern constraints across diverse user profiles and database configurations.

##### 3.1.2.1 Energy Target Adherence

Weekly energy totals track target values closely across all databases, as shown in Table 5. The overall mean relative difference is −3.08% with strong agreement between generated and target values (*r* = 0.935). Among the individual databases, Spanish shows the smallest overall deviation (+0.40%; *r* = 0.996), whereas Irish and Hungarian exhibit modest underestimation (−3.88% and −2.51%, respectively). Across evaluation strata, most relative differences remain within approximately ±5%. Larger deviations are observed for Irish vegan dietary preference and dairy allergy strata, reaching approximately −11% to −12%, and are accompanied by lower correlation values.

**Table 5:** Energy recommendation performance of generated weekly meal plans across databases and evaluation strata.

		Irish database			Spanish database			Hungarian database			All databases		
Stratum	n	Mean ±SD [%] (95% CI)	r	*p*	Mean ±SD [%] (95% CI)	r	*p*	Mean ±SD [%] (95% CI)	r	*p*	Mean ±SD [%] (95% CI)	r	*p*
*Season*													
Winter	1000	−3.90 ± 6.35 (-4.29, -3.51)	0.902	< 0.001	+0.86 ± 3.31 (+0.65, +1.07)	0.994	< 0.001	−2.46 ± 5.09 (-2.78, -2.14)	0.939	< 0.001	−3.08 ± 5.74 (-3.44, -2.72)	0.933	< 0.001
Spring	1000	−3.90 ± 6.44 (-4.30, -3.50)	0.897	< 0.001	−0.05 ± 1.08 (-0.12, +0.02)	0.999	< 0.001	−2.40 ± 5.04 (-2.71, -2.09)	0.939	< 0.001	−3.09 ± 5.69 (-3.44, -2.74)	0.936	< 0.001
Summer	1000	−3.91 ± 6.46 (-4.31, -3.51)	0.898	< 0.001	−0.07 ± 0.91 (-0.13, -0.01)	0.999	< 0.001	−2.66 ± 5.41 (-3.00, -2.32)	0.929	< 0.001	−3.16 ± 5.83 (-3.52, -2.80)	0.932	< 0.001
Autumn	1000	−3.80 ± 6.37 (-4.20, -3.40)	0.900	< 0.001	+0.87 ± 3.25 (+0.67, +1.07)	0.994	< 0.001	−2.53 ± 5.21 (-2.85, -2.21)	0.935	< 0.001	−3.01 ± 5.63 (-3.36, -2.66)	0.937	< 0.001
*Preference*													
Omnivore	3552	−3.72 ± 6.16 (-3.92, -3.52)	0.909	< 0.001	+0.41 ± 2.47 (+0.33, +0.49)	0.996	< 0.001	−2.38 ± 5.09 (-2.55, -2.21)	0.936	< 0.001	−3.11 ± 5.77 (-3.30, -2.92)	0.934	< 0.001
Pescatarian	120	−4.51 ± 7.91 (-5.94, -3.08)	0.866	< 0.001	+0.61 ± 2.45 (+0.17, +1.05)	0.996	= 0.038	−3.10 ± 5.90 (-4.17, -2.03)	0.935	< 0.001	−4.34 ± 6.99 (-5.60, -3.08)	0.917	< 0.001
Vegetarian	280	−3.73 ± 6.16 (-4.45, -3.01)	0.919	< 0.001	+0.20 ± 2.36 (-0.08, +0.48)	0.995	= 0.952	−3.20 ± 5.02 (-3.79, -2.61)	0.951	< 0.001	−2.37 ± 4.57 (-2.91, -1.83)	0.953	< 0.001
Vegan	72	−10.96 ± 10.87 (-13.51, -8.41)	0.582	< 0.001	+0.21 ± 2.62 (-0.41, +0.83)	0.996	= 0.690	−5.53 ± 7.66 (-7.33, -3.73)	0.899	< 0.001	−2.47 ± 4.32 (-3.49, -1.45)	0.958	< 0.001
*Allergy*													
None	3664	−3.25 ± 5.38 (-3.42, -3.08)	0.936	< 0.001	+0.42 ± 2.55 (+0.34, +0.50)	0.996	< 0.001	−2.39 ± 5.00 (-2.55, -2.23)	0.940	< 0.001	−2.93 ± 5.53 (-3.11, -2.75)	0.938	< 0.001
Dairy allergy	264	−12.24 ± 11.42 (-13.62, -10.86)	0.750	< 0.001	+0.30 ± 1.53 (+0.11, +0.49)	0.998	< 0.01	−3.78 ± 6.83 (-4.61, -2.95)	0.905	< 0.001	−5.00 ± 7.45 (-5.90, -4.10)	0.898	< 0.001
Nuts allergy	96	−4.62 ± 6.96 (-6.03, -3.21)	0.920	< 0.001	+0.04 ± 0.77 (-0.12, +0.20)	0.999	= 0.987	−3.66 ± 6.42 (-4.96, -2.36)	0.895	< 0.001	−3.75 ± 6.29 (-5.02, -2.48)	0.943	< 0.001
Overall	4024	−3.88 ± 6.40 (-4.08, -3.68)	0.899	< 0.001	+0.40 ± 2.47 (+0.32, +0.48)	0.996	< 0.001	−2.51 ± 5.19 (-2.67, -2.35)	0.935	< 0.001	−3.08 ± 5.72 (-3.26, -2.90)	0.935	< 0.001

Values show *n*, mean relative difference ± SD and corresponding 95% confidence interval (CI), Pearson correlation (*r*), and one-sample *t*-test *p*-value.

**Figure 2:**
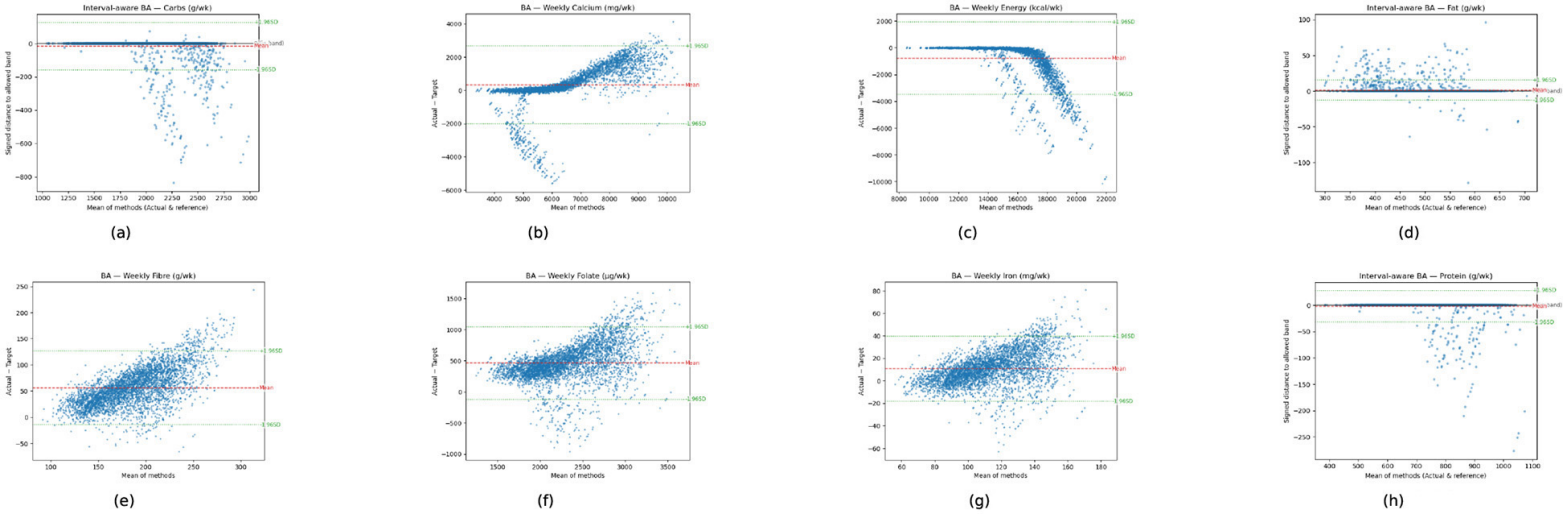
Bland–Altman diagnostic panels for eight nutritional metrics in the Irish database. **(a)** Carbs. **(b)** Calcium. **(c)** Energy. **(d)** Fat. **(e)** Fiber. **(f)** Folate. **(g)** Iron. **(h)** Protein.

The energy panel of the Bland–Altman diagnostics in Figure 2 further illustrates the agreement between generated and target weekly energy values. Weekly energy differences remain concentrated near zero across most of the observed range, with larger negative deviations appearing at higher weekly target values. Most observations remain within the ±1.96 SD agreement limits. For clarity, only the *Irish* dataset is shown.

**Figure 3:**
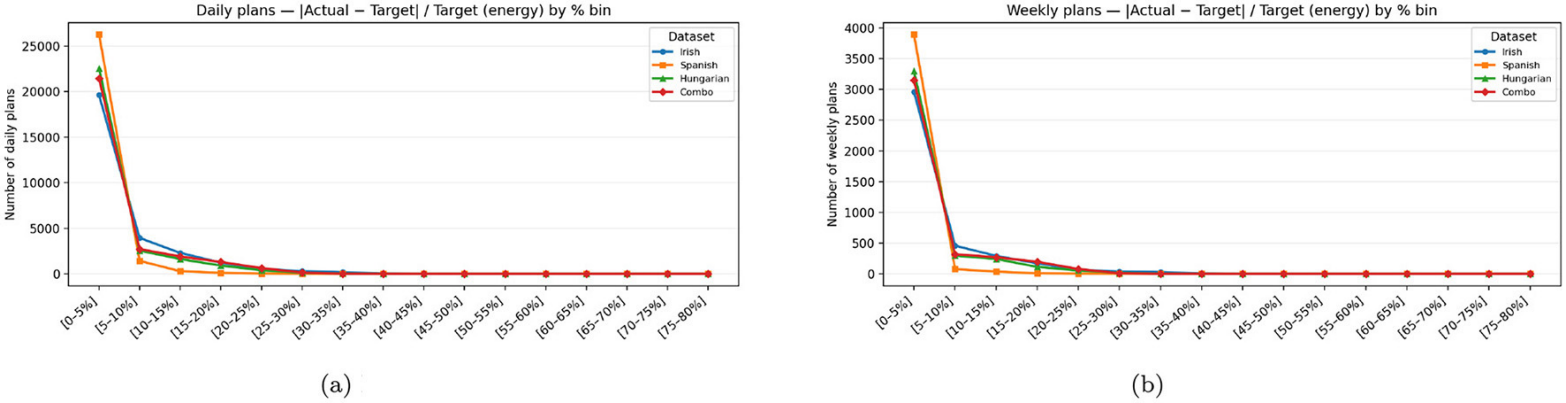
Distribution of relative energy errors across percentage intervals. **(a)** Daily. **(b)** Weekly.

Figures 3a, b show the distribution of daily and weekly energy errors, respectively, across all databases. Most observations fall within the [0–5%] error interval, with frequencies decreasing progressively for larger error ranges. Only a small number of observations exceed the higher error bands.

**Figure 4:**
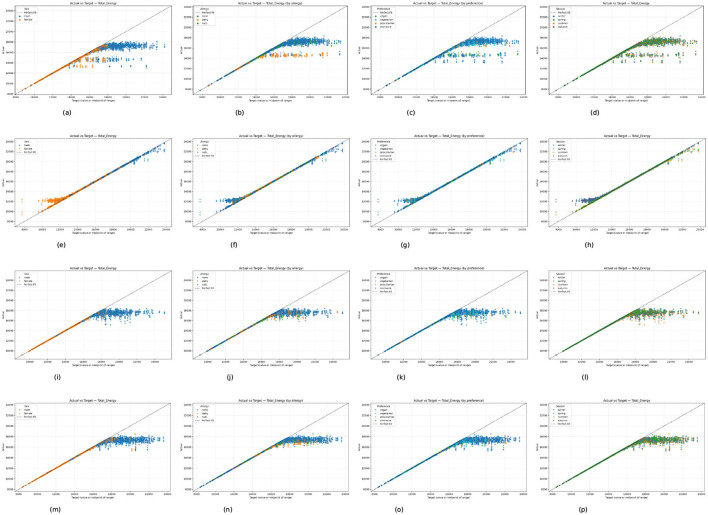
Weekly energy adherence across evaluation strata and databases. Each panel compares generated weekly energy values against per-profile targets; points near the identity line indicate accurate recommendations. **(a)** Irish: by sex. **(b)** Irish: by allergy. **(c)** Irish: by preference. **(d)** Irish: by season. **(e)** Spanish: by sex. **(f)** Spanish: by allergy. **(g)** Spanish: by preference. **(h)** Spanish: by season. **(i)** Hungarian: by sex. **(j)** Hungarian: by allergy. **(k)** Hungarian: by preference. **(l)** Hungarian: by season. **(m)** All datasets: by sex. **(n)** All datasets: by allergy. **(o)** All datasets: by preference. **(p)** All datasets: by season.

Figure 4 shows the relationship between generated and target weekly energy values across databases and evaluation strata. In each panel, the diagonal identity line represents perfect agreement between generated and target energy totals. Most observations closely follow this line, indicating strong agreement across the evaluated configurations. The Spanish database exhibits the tightest clustering around the identity line across the full target range, whereas the Irish, Hungarian, and combined datasets show increasing underestimation at higher target values. Across strata, dairy-allergy and vegan profiles exhibit the largest deviations from the identity line, while omnivore and nuts-allergy profiles remain more closely concentrated around it. Seasonal panels display substantial overlap, indicating relatively small seasonal variation.

##### 3.1.2.2 Nutrient target adherence

Table 6 reports recommendation accuracy for macronutrient targets (protein, fat, and carbohydrate; Macro) and other nutritional targets (fiber, folate, iron, and calcium; Other) across evaluation strata and databases. Overall accuracy averages approximately 70% for macronutrient targets and 63% for micronutrient-related targets. The recommender achieves the highest macronutrient accuracy on the Irish database (~81%), whereas the highest micronutrient-related accuracy is observed on the Hungarian database (~66%); the Spanish database exhibits the lowest macronutrient accuracy (~51%). Across evaluation strata, seasonal variation remains limited, whereas larger deviations appear for constrained dietary profiles, particularly vegan, vegetarian, and milk-allergy strata. Female profiles consistently exhibit higher micronutrient-related accuracy than male profiles across all databases.

**Table 6:** Accuracy (%) of generated meal plans with respect to macronutrient targets (protein, fat, and carbohydrate; “Macro”) and other nutritional targets (fiber, folate, iron, and calcium; “Other”) across databases and evaluation strata.

	Irish		Spanish		Hungarian		All databases	
Stratum	Macro	Other	Macro	Other	Macro	Other	Macro	Other
Season								
Winter	80.50	55.82	53.71	57.02	60.70	65.01	68.97	62.48
Spring	80.12	56.11	49.30	58.07	61.32	64.65	69.31	62.07
Summer	81.23	55.67	48.21	57.99	66.23	66.81	71.97	63.16
Autumn	80.55	56.30	53.48	57.38	62.42	66.54	69.42	63.34
Preference								
Omnivore	81.13	56.46	52.00	57.72	62.30	66.46	69.93	63.43
Pescatarian	77.26	57.95	55.00	57.62	67.47	64.73	70.86	59.20
Vegetarian	79.41	53.21	41.10	55.56	65.65	60.93	71.10	58.55
Vegan	64.58	39.68	43.50	60.32	61.06	51.04	63.00	52.03
Allergy								
None	81.45	56.47	50.87	57.71	63.46	65.89	70.51	63.05
Dairy allergy	69.76	50.28	54.07	55.89	55.95	62.17	60.48	59.19
Nuts allergy	82.11	52.98	53.35	58.15	55.28	70.76	73.10	62.95
Dairy & Nuts	61.83	49.63	57.29	57.22	47.40	61.46	60.34	56.77
Sex								
Male	81.47	48.00	49.52	55.48	57.36	56.68	67.89	53.57
Female	79.73	63.95	52.83	59.75	67.97	74.82	71.94	71.95
Overall	80.60	55.97	51.18	57.61	62.67	65.75	69.92	62.76

The remaining Bland–Altman panels in Figure 2 illustrate agreement between generated and target weekly nutrient values across macronutrient and micronutrient features. For attributes defined by ranges rather than point targets (carbohydrates, fat, and protein), an interval-aware variant is used. Carbohydrates and protein exhibit larger deviations at higher weekly target values, whereas fat deviations remain narrowly distributed around zero. Fiber and folate show comparatively wider dispersion, while iron and calcium remain more tightly concentrated around the target region. Across all nutrient-related attributes, most observations remain within the ±1.96 SD agreement limits.

##### 3.1.2.3 Food-group rule adherence

**Figure 5:**
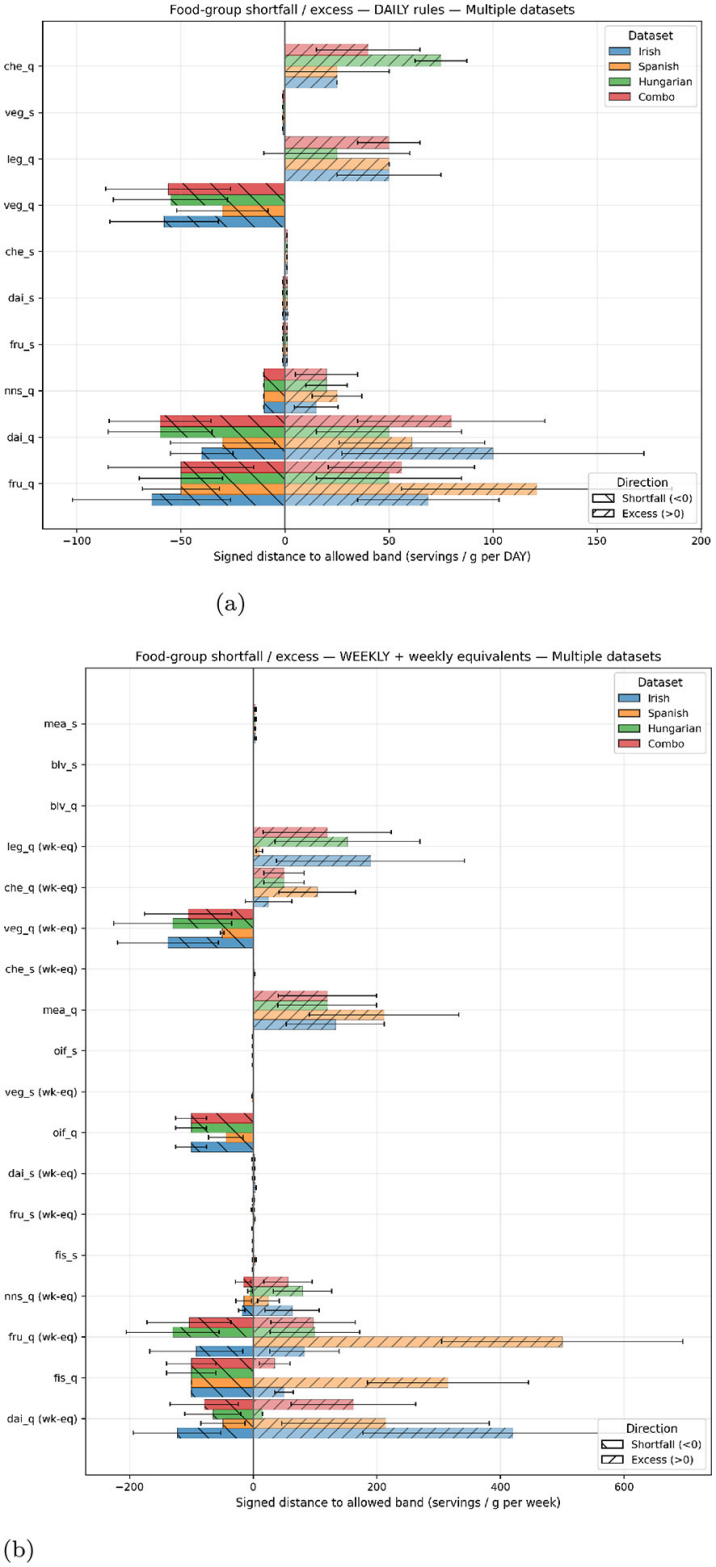
Shortfall and excess distributions for food-group rules (Irish dataset). Y-axis labels denote food groups and units; g = grams, s = servings. **(a)** Daily food-group rules. **(b)** Weekly food-group rules and weekly equivalents of daily rules.

Figure 5 shows the distribution of shortfalls and excesses for daily and weekly food-group rules, respectively. Daily rules exhibit persistent shortfalls for vegetable grams and, to a lesser extent, fruit grams, whereas dairy grams commonly exceed target values. Similar patterns appear at the weekly level, where vegetable and oily-fish targets tend to remain below target ranges while several weekly serving limits remain comparatively well controlled.

**Figure 6:**
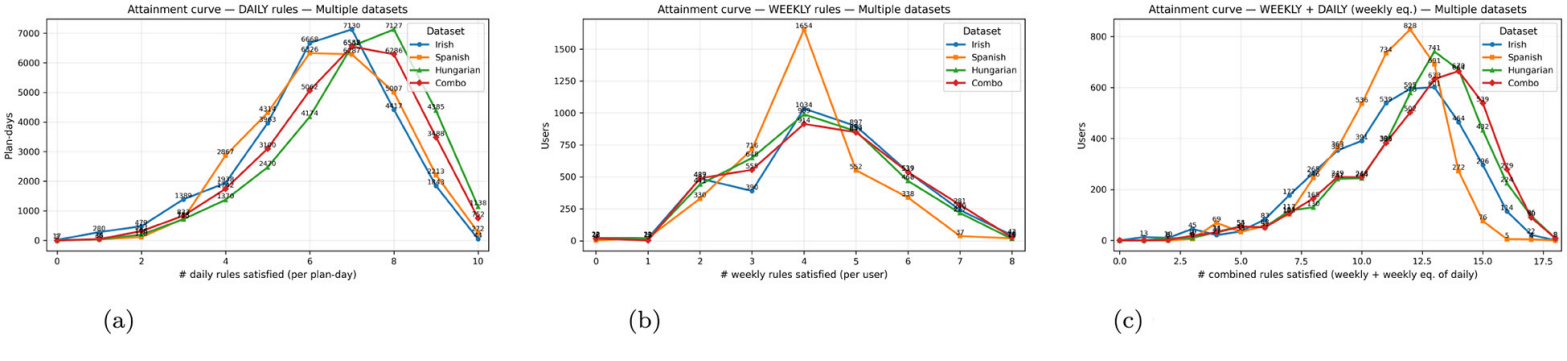
Distributions of simultaneously satisfied food-group rules across databases. **(a)** Daily-rule attainment. **(b)** Weekly-rule attainment. **(c)** Combined daily- and weekly-rule attainment.

Figure 6 further summarizes the distributions of simultaneously satisfied food-group rules across databases. Daily-rule attainment distributions peak around 7–8 satisfied rules per plan-day, whereas weekly-rule attainment peaks around 4 satisfied rules per user-week. When daily and weekly rules are combined, the distributions peak between approximately 12 and 14 simultaneously satisfied rules, with relatively similar shapes across databases.

### 3.2 Descriptinve evaluation of the proposed recommender and state-of-the-art LLMs

To provide a preliminary descriptive assessment of the proposed recommender and contemporary Large Language Models (LLMs) on the meal planning task, we designed two complementary evaluation settings that represent different approaches to constrained meal-plan generation. The first adopts a Retrieval-Augmented Generation (RAG) strategy, in which meal plans are generated only from meals explicitly retrieved from our curated database to ensure rule compliance and minimize hallucinations. The second explores Supervised Fine-Tuning (SFT), assessing whether an instruction-tuned LLM can learn to generate meal plans that conform to our schema and approximate user-specific nutritional targets without external retrieval at inference time. Together, these experiments evaluate the extent to which contemporary LLMs can reproduce the precision, constraint handling, and consistency of the proposed recommender. Both the RAG and SFT evaluations were performed using the open-source LLaMA and Mistral model families.

#### 3.2.1 Retrieval-Augmented Generation (RAG) evaluation

Under the Retrieval-Augmented Generation (RAG) setting (50), weekly meal plans were generated exclusively from items retrieved from the PLAN'EAT Meal Database. Retrieval was restricted to curated meal entries in order to keep generation within a finite and auditable search space while enforcing dietary and output-format requirements.

##### 3.2.1.1 Experimental Setup

We sampled *n* = 35 meals per meal type from the Spanish, Hungarian, and Irish datasets to construct the contextual retrieval pool. Evaluation was performed using ten synthetic omnivorous user profiles without allergies, representing diverse energy requirements (1,500–3,000 kcal) and macronutrient distributions.

##### 3.2.1.2 Generation Procedure

Each user profile was used for meal plan generation through prompts combining a concise few-shot example with explicit structural and numerical constraints. The prompts enforced exclusive use of food-items contained in the retrieved context and prevented the generation of unsupported meal items or extraneous text.

#### 3.2.2 Supervised Fine-Tuning (SFT) evaluation

Under the Supervised Fine-Tuning (SFT) setting, LLMs were fine-tuned on weekly meal plans generated by the proposed recommender in order to generate meal plans directly from user-profile inputs without external retrieval during inference.

##### 3.2.2.1 Training data and experimental setup

We constructed an SFT dataset containing 5,000 Alpaca-style JSON (51) examples per cuisine. Each example paired a synthetic user profile (daily energy target and macronutrient ranges) with the corresponding weekly meal plan generated by the proposed recommender. Weekly plans contained seven daily menus with five meals per day (breakfast, morning snack, lunch, afternoon snack, and dinner), annotated with nutritional information including energy, protein, carbohydrates, and fat.

##### 3.2.2.2 Fine-tuning procedure

Meta-Llama-3.1-8B (52) and Mistral-7B (53) models were fine-tuned using the Unsloth framework (HF/TRL/PEFT) (54) with LoRA-based parameter-efficient adaptation (55). LoRA adaptation employed rank *r* = 16 and scaling factor α = 16 across the standard attention and projection layers. Training employed a context length of 2,048, batch size 2, gradient accumulation 4, three epochs, and a learning rate of 1 × 10^−4^. Optimisation used AdamW-8bit with linear scheduling, weight decay 0.01, automatic FP16/BF16 precision, and EOS-token-based termination.

#### 3.2.3 Descriptive evaluation results

The following evaluation is intended as a preliminary descriptive assessment of the behavior of the examined RAG and SFT approaches under the same meal-planning task and should not be interpreted as a definitive comparative benchmark or as evidence of statistical superiority of one approach over another.

The PLAN'EAT recommender and the RAG and SFT configurations were evaluated using the same ten synthetic omnivorous user profiles without allergies to ensure direct comparability. Accuracy (%) was computed separately for energy (±250 kcal tolerance), protein, carbohydrates, and fat according to their respective target intervals. For each generated plan, deviations from target values were summarized using mean absolute error (MAE) and standard deviation metrics. The comparative evaluation focused on energy and macronutrient adherence, as these variables were explicitly represented in both the RAG prompting framework and the SFT training schema. Micronutrient-level comparison was not included, since generated LLM outputs do not consistently provide the standardized food-item-level nutritional structure required for reliable micronutrient estimation and validation.

Table 7 summarizes the descriptive performance characteristics of the evaluated approaches across the Irish, Spanish, and Hungarian datasets. Accuracy is reported separately for energy, protein, carbohydrates, and fat, together with the corresponding mean absolute errors and standard deviations.

**Table 7:** Accuracy (%), mean absolute error (MAE), and standard deviation for energy and macronutrient targets by model and cuisine.

	Accuracy (%)				MAE				SD			
Model and cuisine	Energy	Protein	Carbs	Fat	Energy	Protein	Carbs	Fat	Energy	Protein	Carbs	Fat
Irish												
RAG–Llama	10.00	80.00	100.00	70.00	644.19	3.65	0.00	3.64	367.61	7.83	0.00	6.11
RAG–Mistral	40.00	80.00	40.00	60.00	478.31	2.09	22.79	1.94	375.79	4.43	33.04	4.42
FT–Llama	50.00	80.00	90.00	80.00	333.18	0.63	12.16	0.71	394.95	1.93	20.17	1.90
FT–Mistral	50.00	80.00	90.00	70.00	257.65	1.04	4.16	2.52	297.81	2.39	13.15	4.36
PLAN'EAT RS	80.00	100.00	90.00	100.00	80.62	0.00	0.66	0.00	173.43	0.00	2.10	0.00
Spanish												
RAG–Llama	20.00	60.00	40.00	20.00	478.57	4.56	28.54	36.57	486.55	7.72	35.27	23.50
RAG–Mistral	30.00	70.00	20.00	20.00	459.86	3.34	29.93	32.01	477.80	6.85	37.75	18.18
FT–Llama	80.00	80.00	80.00	30.00	185.76	1.27	1.32	6.97	191.19	2.75	3.11	8.62
FT–Mistral	30.00	50.00	30.00	30.00	479.73	6.57	16.82	23.18	324.09	8.57	23.18	19.70
PLAN'EAT RS	100.00	100.00	80.00	60.00	1.36	0.00	0.46	1.91	3.84	0.00	1.08	3.60
Hungarian												
RAG–Llama	20.00	20.00	10.00	70.00	597.27	13.16	60.62	1.25	504.24	18.11	55.79	2.35
RAG–Mistral	20.00	20.00	80.00	80.00	611.08	16.15	54.64	1.74	484.29	17.91	46.39	4.58
FT–Llama	70.00	80.00	80.00	90.00	194.43	1.49	12.97	0.44	268.83	3.19	26.93	1.38
FT–Mistral	70.00	70.00	70.00	90.00	205.15	3.77	9.91	0.29	268.92	6.82	19.40	0.90
PLAN'EAT RS	80.00	80.00	80.00	70.00	70.47	1.06	8.63	2.82	151.29	2.45	18.50	5.21

Across the evaluated datasets, the fine-tuned models generally exhibited higher energy and macronutrient adherence than their corresponding RAG configurations. The highest accuracies among the LLM-based approaches are typically achieved by the fine-tuned LLaMA models, whereas performance varies more substantially across cuisines for both RAG and SFT configurations. Within the evaluated experimental setting, the proposed PLAN'EAT recommender exhibited lower energy errors and more stable overall performance across the reported nutritional metrics.

## 4 Discussion

### 4.1 Nutritional and food-group adherence

Before discussing the recommendation results in detail, it is important to note that the three country specific meal datasets differ substantially in their size, coverage, and nutritional composition. The Irish dataset contains substantially fewer meals overall than the Spanish and Hungarian datasets (101 vs. 150 meals), while the number of meals available under dietary restrictions varies considerably, particularly for vegan users (37 meals in the Irish dataset compared with 77 in the Hungarian dataset). Furthermore, the datasets differ markedly in their food-group representation. For example, the Spanish dataset contains substantially more fish and oily-fish options (33 and 10 cumulative servings, respectively, compared with 3 and 4 in the Irish dataset and 2 and 4 in the Hungarian dataset), together with broader representation of vegetables and fruits, all of which are central components of the SHD-derived food-group recommendations used in this study. Such differences influence both the diversity of feasible meal combinations and the optimizer's ability to simultaneously satisfy energy, nutrient, food-group, and variety constraints. Consequently, they provide important context for interpreting the observed variation in recommendation performance across databases.

The energy-adherence results presented in Table 5 demonstrate that the proposed recommendation methodology can closely satisfy personalized weekly energy targets across a broad range of user profiles and database configurations. The strong agreement observed across most evaluation strata suggests that the optimization framework remains stable under varying seasonal, demographic, and dietary conditions. The largest deviations consistently occur in highly constrained cases, particularly vegan and dairy-allergy strata within the Irish database. A likely explanation is the substantially smaller number of feasible meal combinations available for these profiles, which reduces the search space and limits the system's ability to simultaneously satisfy energy, nutrient, and diversity constraints. This interpretation is further supported by the comparatively smaller catalog sizes associated with restrictive dietary conditions in the Irish database.

The Bland–Altman analyses in Figure 2 further reveal that deviations increase primarily at the upper ends of the target distributions, particularly for weekly energy, carbohydrates, and protein. Similarly, the stratified energy scatter plots in Figure 4 indicate that recommendation difficulty increases progressively at higher weekly energy targets, especially in the Irish and Hungarian datasets. The visible flattening below the identity line suggests that highly demanding nutritional profiles reduce the availability of feasible meal combinations capable of simultaneously satisfying nutritional and diversity constraints. In contrast, the Spanish dataset maintains close adherence across the full target range, suggesting that broader meal coverage and richer representation of SHD-relevant food groups may improve robustness under constrained optimization scenarios.

The error distributions shown in Figure 3 provide additional evidence of the recommender's stability. The large concentration of observations within the lowest error bins indicates that accurate recommendations are achieved for the majority of generated plans, while the relatively small number of large-error cases suggests that substantial deviations arise primarily under highly constrained recommendation scenarios rather than from systematic model bias or calibration failure.

Beyond energy targets, the nutrient-adherence results reported in Table 6 indicate that the recommender satisfies macronutrient targets more consistently than the other nutritional targets considered in this study (fiber, folate, iron, and calcium). A contributing factor may be that the optimization process must simultaneously satisfy food-group constraints derived from SHD guidelines, which encourage lower consumption of some animal-source food-items while maintaining EFSA-based micronutrient targets. This may reduce the number of nutritionally feasible meal combinations, particularly for restrictive dietary patterns. Performance differences across dietary-preference and allergy strata are especially pronounced for vegan, vegetarian, and dairy-allergy profiles, which substantially reduce the number of feasible meal options available to the optimization process. In contrast, omnivore profiles remain comparatively stable across databases. The comparatively lower macronutrient accuracy observed in the Spanish dataset may further suggest that differences in meal-catalog composition, food-group representation, and nutrient distributions influence optimization feasibility. To further investigate this observation, we analyzed the nutritional characteristics of 100,000 randomly generated daily plans from each national dataset and compared them with the target ranges derived from the synthetic user profiles. While average protein content in the Spanish recommendation space (113 g/day) was generally compatible with the corresponding target range (approximately 84–141 g/day), average carbohydrate content (229 g/day) fell slightly below the target range (approximately 253–338 g/day), and average fat content (118 g/day) substantially exceeded the target range (approximately 50–88 g/day). The elevated fat levels were also markedly higher than those observed in the Hungarian (67 g/day) and Irish (48 g/day) recommendation spaces. This pattern is consistent with the food-group composition of the Spanish dataset, which contains larger quantities of fish, meat, dairy, and cheese than the other national datasets. Together, these findings suggest that the reduced macronutrient accuracy is primarily associated with difficulties in satisfying fat targets rather than a uniform inability to satisfy all macronutrient requirements. Finally, the consistently higher accuracy observed for female profiles may reflect the lower average energy targets associated with these profiles, which reduce optimization pressure across competing nutritional constraints.

The food-group adherence analyses presented in Figures 5, 6 indicate that some dietary rules are systematically more difficult to satisfy simultaneously than others. In particular, the persistent vegetable and oily-fish shortfalls suggest that these targets compete strongly with other nutritional and diversity constraints within the available meal catalogs. Conversely, the comparatively stable attainment of several weekly serving limits indicates that frequency-based constraints are easier to accommodate during optimization than quantity-based nutritional targets. The broadly similar attainment distributions observed across databases further suggest that the recommender exhibits relatively stable rule-balancing behavior despite the substantial differences in catalog composition and meal availability across databases.

Overall, these findings indicate that recommendation difficulty is not uniform across nutritional objectives and food-group rules, and increases substantially in regions where multiple nutritional, diversity, dietary, and database-composition constraints must be satisfied simultaneously.

### 4.2 Comparison with state-of-the-art LLMs

The comparative results in Table 7 suggest that supervised fine-tuning substantially improves the energy and macronutrient adherence accuracy of LLM-generated meal plans relative to retrieval-augmented generation alone, with the largest gains observed for energy adherence. However, even when trained directly on meal plans produced by the proposed recommender, the fine-tuned models remain less consistent than PLAN'EAT, particularly with respect to energy error and overall variability. Although some fine-tuned models achieve macronutrient accuracies comparable to the recommender in specific datasets, their error magnitudes remain substantially larger. This indicates that learning the statistical structure of recommender outputs is insufficient to fully reproduce the explicit constraint satisfaction achieved by the underlying optimization process. The results suggest differences in the ability of the evaluated approaches to satisfy nutritional targets under the experimental conditions considered.

### 4.3 Strengths, limitations, and future work

An important strength of the proposed framework is its transparency and explainability. Recommendations are generated through explicit nutritional targets, food-group constraints, and interpretable scoring functions, allowing the factors influencing meal-plan selection to be inspected and traced to specific dietary requirements and guideline-based rules. This contrasts with many generative AI approaches, where the rationale underlying individual recommendations may be less transparent. Such transparency may support user trust and facilitate clinical acceptability, as nutrition professionals can more readily verify, audit, and communicate the basis of generated recommendations.

The present study focuses on technical validation of the proposed recommendation framework through large-scale *in-silico* experiments using synthetic user profiles. While this approach enables systematic assessment of nutritional adherence, scalability, and constraint satisfaction across a wide range of recommendation scenarios, it does not provide evidence regarding usability, user acceptance, adherence to recommendations, behavioral change, or long-term health outcomes. Consequently, the findings should be interpreted as a technical validation of the recommendation framework rather than a demonstration of real-world effectiveness.

Several additional limitations should also be acknowledged. *First*, sustainability was represented indirectly through SHD-derived food-group recommendations adapted from the MyPlanetDiet framework (46, 47), which promotes dietary patterns associated with lower environmental impact, including reduced consumption of red and processed meat and increased consumption of plant-based foods. However, environmental sustainability was not directly quantified through explicit indicators such as greenhouse-gas emissions, water use, or land-use metrics. *Second*, the current optimization framework considers only a subset of micronutrients (folate, calcium, and iron), while other nutritionally relevant micronutrients, including vitamin B12, vitamin C, zinc, iodine, magnesium, and potassium, are not explicitly modeled. As a result, meal plans that satisfy the optimization objectives may still exhibit deficiencies in untracked micronutrients, particularly for more restrictive dietary patterns such as vegetarian and vegan diets, where some of these nutrients are of greater concern. *Third*, allergen support is currently limited to dairy and nuts and therefore does not provide comprehensive coverage of the 14 major allergens recognized under EU food-labeling regulations. Consequently, the present evaluation does not constitute evidence that the framework can safely accommodate individuals with allergen requirements beyond those explicitly modeled in this study. Nevertheless, the underlying recommendation framework is designed to support additional allergen constraints, subject to the availability and completeness of the corresponding allergen information in the underlying datasets. *Finally*, the descriptive evaluation against state-of-the-art LLMs should be interpreted within the scope of its design and should not be considered a definitive benchmark of recommender-system and LLM performance. In particular, the SFT models were trained on meal plans generated by the proposed recommender, which may favor the reproduction of recommendation patterns present in the training data. Consequently, the reported results should be interpreted as an exploratory assessment of the ability of contemporary LLMs to reproduce nutritionally constrained meal-planning recommendations under the evaluated conditions.

Future work will focus on evaluating the framework with real users under real-life conditions to assess user engagement, acceptance, adherence to recommendations, and potential dietary behavior changes over longer periods. Additional developments will include expanding meal coverage, incorporating additional micronutrients and allergen constraints, integrating explicit environmental sustainability and cost-related objectives, enhancing explanation capabilities, and incorporating user-feedback mechanisms.

## 5 Conclusion

This paper presented the PLAN'EAT Nutrition Advisor, an AI-driven recommendation framework for generating personalized weekly meal plans that combines a nutritionist-curated meal database, expert-defined nutritional rules, score-based optimization, and diversity-aware meal selection. Beyond the recommendation methodology itself, the work contributes a harmonized meal database spanning Irish, Spanish, and Hungarian cuisines, developed through a structured curation process to support nutritionally balanced and culturally relevant meal planning.

The proposed framework was evaluated through a large-scale *in-silico* validation study involving 1,000 synthetic user profiles across multiple seasons, dietary preferences, allergies, demographic characteristics, and database configurations. The results demonstrate that PLAN'EAT can satisfy personalized weekly energy targets with high accuracy while maintaining strong adherence to macronutrient, micronutrient, and food-group objectives across a wide range of recommendation scenarios. Although recommendation difficulty increases for highly constrained profiles and demanding nutritional targets, the system remains robust across databases and user strata. The analyses further show that balancing multiple nutritional, dietary, diversity, and SHD-derived food-group constraints simultaneously is feasible within a transparent optimization-based recommendation process.

A comparative evaluation against state-of-the-art open-source LLMs further demonstrated that supervised fine-tuning substantially improves nutritional recommendation quality relative to retrieval-augmented generation. Nevertheless, the proposed recommender exhibited lower errors and more stable energy and macronutrient adherence than the evaluated retrieval-augmented and fine-tuned models. These findings should be interpreted descriptively and suggest that the evaluated generative approaches did not fully reproduce the level of nutritional adherence achieved by the proposed framework under the experimental conditions considered.

Overall, the findings highlight the value of combining expert nutritional knowledge, curated food data, and transparent optimization techniques for personalized nutrition recommendation. To promote transparency, reproducibility, and future research, the PLAN'EAT Meal Database is publicly available through Zenodo (56). Future work will focus on extending and evaluating the framework in real-world settings, broadening its nutritional, allergen, sustainability, and cost-related scope, and enhancing its personalization and explanation capabilities. In addition, future research should investigate how meal-database composition and coverage influence optimization performance under complex nutritional and dietary constraints.

## Data Availability

The raw data supporting the conclusions of this article will be made available by the authors, without undue reservation.
